# Diabetes Prevention: Knowledge and Perception of Risk among Italian Population

**DOI:** 10.1155/2019/2753131

**Published:** 2019-10-31

**Authors:** Concetta P. Pelullo, Riccardo Rossiello, Roberto Nappi, Francesco Napolitano, Gabriella Di Giuseppe

**Affiliations:** Department of Experimental Medicine, University of Campania “Luigi Vanvitelli”, Naples 80138, Italy

## Abstract

The risk perception for developing diabetes has not been well established. The aim of this study is to evaluate knowledge and perception of risk for developing diabetes. A cross-sectional study was conducted among a sample of 527 parents of children attending public schools in Naples (Italy). A self-administered anonymous questionnaire was used to collect the data. In total, 97.3% of participants have heard about diabetes, but only 16.7% knew the main risk and protective factors. This knowledge was statistically significantly higher in those who had close relatives with diabetes. Moreover, those who had middle school or lower and high school education, compared with those who had a college degree or higher, were less knowledgeable. The mean total value of the risk perception for developing diabetes was 1.9. Females those who had more than 40 years of age, those who needed of additional information, those who had a higher BMI, those who had close relatives with diabetes, those who had at least one chronic disease, and those who reported a lower value of self-rated health status were more likely to perceive a higher risk for developing diabetes. Moreover, this perception was statistically significantly lower among those who had a middle school or lower and high school education, compared with those who had a college degree or higher. The knowledge about diabetes needs to be improved, and the low risk perception for developing diabetes among the sample is worrying given the severity of the disease and the preventive measures available.

## 1. Introduction

It is well known that diabetes is one of the most common noncommunicable diseases in the world, and it is a serious global health burden with a large economic impact at multiple levels [1]. Worldwide, diabetes kills about 3.4 million people annually, and in Europe, there are about 60 million people with diabetes, and this prevalence is increasing among all ages, mostly due to increases in overweight and obesity, unhealthy diet, and physical inactivity [2]. In Italy, approximately three million people had diabetes, and this number is expected to rise to more than four million by 2035 [3, 4].

Despite evidence that diabetes can be delayed or prevented, the lifestyle change remains a major challenge, and people of all ages should achieve and maintain healthy body weight, be physically active, eat a healthy diet, and avoid tobacco use. Adapting to a healthier lifestyle requires the implementation of behavioral changes, and the perception of risk is believed to be important determinant of preventive health behaviors [5–7].

Relatively poor is the research about the public knowledge and risk perception for developing diabetes. Previous epidemiological studies have been conducted to evaluate the diabetes risk perception in pharmacists [8], foreign-born Spanish-speaking US Latinos [9], primary care patients [10, 11], and other different populations [12–14], but to our knowledge, no research has been conducted in Italy on this issue. Understanding the public diabetes knowledge and the risk perception for developing diabetes is imperative in order to plan effective educational interventions for its prevention and increase awareness of the health risks of this disease. Therefore, this investigation has three objectives: (1) to evaluate knowledge about diabetes; (2) to assess the perception of risk for developing diabetes; and (3) to determine the factors associated with these outcomes of interest.

## 2. Materials and Methods

A cross-sectional survey was conducted from September to November 2016 among a random sample of parents of students attending six public schools (primary, middle, and high schools). The parents were selected through a two-stage cluster sampling. In particular, from the list of public schools in the geographic area of Naples, Italy, six schools were randomly selected using a computer-generated list of random numbers, and from each school, the students were selected through a simple random sampling. The selected schools were located in the metropolitan area of Naples, a region with 92 municipalities characterized by a small territorial extension (1179 km^2^) and high population density with more than 3 million people.

The sample size was calculated assuming that 23% of respondents had a high perception level of risk to develop diabetes in accordance with literature [10, 12], a confidence interval of 95%, a maximum error of 5%, a response rate of 60%, and a design effect of two, for a total of 906 participants.

Before starting the study, collaboration and permission to carry out the survey were obtained by the heads of each selected school. Data were collected by a self-administered anonymous questionnaire. The questionnaires were delivered, in a sealed envelope, to the students in each classroom by the research team, and it was requested that the questionnaire was filled by parents. The parents also had received a letter containing information about the purpose of the survey inviting only one parent to complete the questionnaire, where it was explained that their participation was voluntary, that all information gathered would be anonymous, and that confidentiality of information would be maintained by omitting any personal information. An envelope to facilitate the return of the completed questionnaire was made available. Written informed consent for participation was requested before completing the questionnaire. A pilot study was carried out on 30 parents of students attending primary, middle, and high schools, similar to those included in the study and not included in the final sample, in order to evaluate acceptability in terms of length, clarity, and question format. Feedback was incorporated into the survey prior to the start of the investigation.

The questionnaire was divided into five sections. The sociodemographic and anamnestic section focused on personal characteristics of respondents, such as sex, age, nationality, weight and height, marital status, educational level, employment status, number of children, medication use, smoking status, self-reported health status, having diabetes, having close relatives with diabetes, and comorbidity. Moreover, the body mass index (BMI) was calculated from their self-reported weight and height. The self-reported health status of participants was assessed on a 10-point Likert-type scale, with responses ranging from 1 (poor) to 10 (excellent). The knowledge about diabetes was explored with three questions including definition, risk, and protective factors of diabetes. This section provided responses in several formats: “yes” or “no” for the definition and closed-end with categorical (yes or no) for the risk and protective factors.

In the attitude section, the risk perception for developing diabetes has been evaluated by using the composite risk score of the Risk Perception Survey for Developing Diabetes (RPS-DD), a validate questionnaire of 43 items [13], translated in Italian language. In particular, the composite risk score combined 32 items on different subscales including the Personal Disease Risk (15 items), Comparative Environmental Risk (9 items), Optimistic Bias (2 items), Personal Control (4 items), and Worry (2 items). This score was assessed on a 4-point scale, with response ranging from 1 (strongly disagree) to 4 (strongly agree). The higher score indicates greater overall perceived risk related to diabetes and its complications.

The questionnaire was also composed of a section which investigated on dietary and physical activity habits and respondents' test of blood glucose, cholesterol, and blood arterial pressure in the last year. Finally, parents were asked regarding the sources of information about the diabetes and the need of additional information.

Participants with diabetes and under 18 years of age were excluded. Ethical approval of the study protocol and of the survey instrument was obtained from the Ethical Committee of Teaching Hospital of University of Campania “Luigi Vanvitelli.”

### 2.1. Statistical Analysis

Statistical analysis was conducted in multiple stages. First, descriptive analysis was performed, and frequencies and percentages were reported for each characteristic and response of the sample. Then, bivariate analysis was carried out using the *t* test and chi-square test to evaluate the association between potential explanatory variables and each outcome of interest. Then, to account for the two-stage cluster sampling, linear and logistic regression models were estimated by using a Generalized Estimation Equation (GEE) analysis in order to investigate independent characteristics associated with the following outcomes of interest: knowledge of the main risk (relatives with diabetes and obesity) and protective factors (healthy diet, physical activity, and low body weight) of diabetes (no = 0; yes = 1) (Model 1); risk perception for developing diabetes based on RPS-DD composite score (continuous) (Model 2); and worry of getting diabetes, based on the values of worry subscale of RPS-DD (continuous) (Model 3).

In all models, the following independent variables were included: age (≤40 years = 0; >40 years = 1), gender (male = 0; female = 1), marital status (unmarried = 0; married = 1), number of children (one = 0; more than one = 1), educational level (three categories: no formal education/primary/middle school = 1; high school = 2; college degree or higher = 3), employment status (no = 0; yes = 1), BMI (continuous), having at least one chronic disease (no = 0; yes = 1), medication use (no = 0; yes = 1), having close relatives with diabetes (no = 0; yes = 1), smoking status (three categories: no smoker = 1, ex-smoker = 2, smoker = 3), self-reported health status (continuous), physicians as source of information about diabetes (no = 0; yes = 1), and need of additional information about diabetes (no = 0; yes = 1). Moreover, knowledge of the main risk and protective factors of diabetes (no = 0; yes = 1) was included in Models 2 and 3.

The results of multivariate regression analysis were reported as odds ratios (ORs) and 95% confidence intervals (CIs). Standardized regression coefficients (*β*) were reported for the linear regression model. All inferential tests were performed through the execution of bilateral hypothesis test with statistically significant levels for *p* values equal to or less than 0.05. All analysis was performed using Stata version 15 statistical software [15].

## 3. Results

Of 944 questionnaires delivered, 8 were excluded because the respondents had self-reported a diabetes status. Therefore, of the 936 eligible participants, 527 completed the questionnaire for an overall response rate of 56.3%. The main characteristics of respondents are presented in Table 1. More than half of participants (54.3%) were female, the mean age was 44.4 years (range 28–72), almost all were married (91.2%), 83.5% had more than one child, 19.9% had a college degree or higher, and more than half were employed (59.9%). Moreover, a third of sample was smoker (32.6%) and had at least one chronic disease (35.3%), 27.9% had close relatives with diabetes, the mean value of self-reported health status was 7.8, and the mean value of BMI was 25.6.

Regarding the knowledge, almost all the participants (97.3%) reported having heard about diabetes, and 64.5% and 60.9% of them correctly identified familiarity and obesity as risk factors, while 85.6%, 50%, and 32.8% identified, respectively, healthy diet, physical activity, and lower body weight as protective factors. Overall, only 16.7% of participants knew the main risk and protective factors. The results of multivariate logistic regression analysis revealed that those who had close relatives with diabetes compared with those who do not had them (25.4% vs 13.2%; *p*=0.003) were more likely to have this knowledge. Moreover, those who had middle school or lower (6.3% vs 41.3%; *p* < 0.001) and high school education (16.3% vs 41.3%; *p* < 0.001), compared with those who had a college degree or higher, were less knowledgeable (Model 1 in Table 2).

More than two-thirds (69.3%) of participants perceived a slight risk for developing diabetes, with a mean total value of a risk perception of 1.9. The model showed that female participants those who had more than 40 years of age, those who needed of additional information about diabetes, those who had a higher BMI, those who had close relatives with diabetes, those who had at least one chronic disease, and those who reported a lower value of self-rated health status were more likely to perceive a higher risk for developing diabetes. Moreover, this perception was lower among those who had a middle school or lower and high school education (Model 2 in Table 2).

The mean value of the worry for developing diabetes was 2.7, on a scale from 1 to 4. The results of the model showed that respondents with middle school or lower and high school education compared to those who had a college degree or higher and those who needed additional information about diabetes were more likely to have a higher worry for developing diabetes (Model 3 in Table 2).

More than two-third (77.9%) of respondents reported a moderate physical activity, while only 15.7% reported an intense activity. As regards dietary habits, only 10.4% reported vegetable intake 2–3 times a day and 12.3% whole grains once a day. Furthermore, 34.2% consumed red meat 1–4 times a month and 50.6% fish 2–4 times a week. Moreover, 56.1%, 57%, and 64.1% reported to have checked in the last year the blood glucose, cholesterol, and the arterial pressure, respectively.

In terms of sources of information, 87.3% received information, and the most frequent sources reported by participants were in the following order: physicians (35.7%), relatives (33.9%), and television/newspapers (30%). Moreover, 46.1% of respondents indicated that they would receive additional information about diabetes.

## 4. Discussion

This survey provided interesting findings regarding diabetes knowledge and risk perception for developing diabetes among a sample of a nondiabetic participants, and to our knowledge, it represents the first study of this kind carried out in Italy.

For a correct evaluation of this study, it must be considered that the comparison of our results with those of other investigations is difficult due to the different objectives, methodology, groups of populations, and geographical areas considered. In this survey, almost all the respondents have heard about diabetes, but a lower level of participants' knowledge was found regarding the main risk and protective factors of diabetes. Indeed, less than one-fifth have a good knowledge of the main risk and protective factors. The results of this survey regarding the participants' knowledge are very worrying because it is well established that a healthy lifestyle is considered the most effective method to prevent or delay the onset of diabetes, and an inadequate knowledge of the diabetes' causes and complications could negatively affect the access to appropriate preventive measures. However, this low level of knowledge is in line with findings reported in previous studies among different groups of population [16–18].

The respondents' attitudes regarding the risk perception for developing diabetes in this survey indicate an overall perceived slight risk and, moreover, a large majority of the participants are not worried for developing diabetes. Although this study was conducted on the general population whose health status was unknown, the fact that the majority of the sample has a low perception of the risk for developing diabetes must be of concern because the prevalence of this condition in the Italian population is estimated >5% and it increases constantly [19]. It is therefore urgent to plan health education interventions that can increase the public knowledge and the correct perception of the risk for developing diabetes. In particular, the results indicate that more efforts must be made by policy makers and healthcare workers to make the population aware of the fact that the correct lifestyles are an effective preventive measure of diabetes.

The results of the multivariate regression analysis indicated that there are different factors influencing the outcomes of interest. For example, participants who had a college degree or higher level of education were more likely to know the main risk and protective factors of diabetes. Similar findings have been found by other investigations conducted on knowledge regarding the diabetes among different at-risk groups of population [16, 20, 21]. Moreover, previous experiences in the same geographical area of this study confirmed the influence of a higher educational level on the knowledge regarding the health topics [22–25]. In addition, our results showed that respondents that were female, older, suffered at least one chronic disease, and had close relatives with diabetes were more likely to perceive a higher risk for developing diabetes. These findings are in line to those reported in a study conducted in the US among primary care patients [10] and among adult population in Germany [11]. Furthermore, the results of our study show that those with a high BMI had a strong risk perception of diabetes. This finding was similar to that of a study conducted in the US in which a higher BMI was associated with the risk perception for developing diabetes [26]. The results related to the factors influencing the knowledge and the risk perception provide important information to implement targeted preventive interventions on specific groups of the population, since in Italy, the prevalence of diabetics increases with age, and it is more frequent among men, among less educated people, and in the southern regions [27].

In this survey, participants who needed additional information about diabetes were more likely to have a higher worry for developing diabetes. Therefore, there is a clear need of effective public educative actions about diabetes prevention provided by healthcare workers since physicians are the most frequent reported sources of knowledge by our sample. The key role of physician as important sources of knowledge information has been confirmed in other studies previously conducted by some of us [22, 28–30].

This investigation has some limitations to take into account for a correct interpretation of the results. The first limit consists in the design of this study in which it is not possible to determine the causal relationship between the outcomes of interest and predictors. Second, some information as well as the lifestyle habits and clinical checks was measured by the self-reported data of the respondents, and it is possible that a recall bias has occurred and the respondents could report more socially desirable behaviors. Third, the participants' recruitment has been performed through public primary, middle, and high schools, and a part of the younger population as well as parents of students who attended private schools or whose children have graduated was excluded, and therefore the study population cannot be representative of the whole public in the geographic area where the survey was carried out. Fourth, as regards the characteristics of respondents and nonrespondents, we can assume that these are similar because they are equally distributed for the age of the children frequenting the different schools. Finally, although we cannot exclude that our findings pertain only to this area, it is possible to assume that the sociodemographic characteristics of the participants are analogous to those of the population in other regions of Italy. However, this study has several strengths. Indeed, the sample size is large and has been carefully selected, and the response rate is good. Moreover, since very few data are available on knowledge and risk perception about diabetes, we believe that the results of our study can help to improve the knowledge on this topic in Italy.

## 5. Conclusions

This study showed that the knowledge about the diabetes need to be improved, and the low risk perception for developing diabetes among the sample is worrying given the severity of the disease and the preventive measures available. Policy makers, healthcare managers, and healthcare workers should plan more effective educational interventions to increase the public knowledge of diabetes and to improve the lifestyle, adopting healthy behaviors in order to reduce the social and medical burden of diabetes. In particular, the results of our study showed that educational interventions must be directed above all towards the less educated population, also considering that in Italy, about a million people, despite having diabetes, are not aware of it. Furthermore, socioeconomic difficulties are a strong predictor of diabetes given that the prevalence of the diabetes is higher in the lower social classes [31]. Moreover, the findings of this study also indicate that participants who had close family members with diabetes and those with chronic diseases or high BMI were more likely to perceive a higher risk for developing diabetes. Therefore, specific prevention interventions should be targeted at these at-risk groups of population in order to change unhealthy behaviors or implement appropriate early diagnosis measures to avoid long-term complications of the disease.

## Figures and Tables

**Table 1:** Sociodemographic characteristics and selected information about the study population.

	*N*	%
Sex		
Male	241	45.7
Female	286	54.3

Age	44.4 ± 7.7	(28–72)^+^
≤40	164	31.1
>40	363	68.9

Marital status^*∗*^		
Married	480	8.8
Other	46	91.2

Educational level		
No formal education, elementary, or middle school	192	36.8
High school	226	43.3
College degree or higher	109	19.9

Number of children^*∗*^		
1	85	16.5
>1	441	83.5

Employment status^*∗*^		
Unemployed	207	40.1
Employed	309	59.9

BMI^*∗*^		
<25	253	48.1
≥25	274	51.9

Medication use^*∗*^		
No	390	74.3
Yes	135	25.7

Smoking status^*∗*^		
No smokers	246	47.0
Ex-smokers	107	20.4
Smokers	171	32.6

At least one chronic disease^*∗*^		
No	339	64.7
Yes	185	35.3

Having close relatives with diabetes^*∗*^		
No	372	72.1
Yes	144	27.9

Perception of personal health status^*∗*^		
≤7	226	43
≥8	300	57

^+^Mean ± Standard deviation (range). ^*∗*^Number for each item may not add up to total number of study population due to missing value.

**Table 2:** Multivariate logistic and linear regression analysis to characterize factors associated with the different outcomes of interest using a Generalized Estimation Equation (GEE) analysis.

Model 1: knowledge of the main risk and protective factors of diabetes	OR	SE	95% CI	*p* value

Educational level				
College degree or higher	1^*∗*^			
No formal education/elementary/middle school	0.1	0.04	0.05–0.23	<0.001
High school	0.24	0.07	0.13–0.44	<0.001
Who have close relatives with diabetes	2.31	0.65	1.33–4.02	0.003
Age > 40 years	1.99	0.71	0.99–3.99	0.052
Smoking status				
Smokers	1^*∗*^			
No smokers	1.74	0.57	0.92–3.33	0.088
Ex-smokers	1.62	0.64	0.74–3.51	0.225
Who have at least a chronic disease	1.68	0.55	0.88–3.19	0.111
Perception of personal health status	0.66	0.19	0.38–1.14	0.142
Female	1.58	0.51	0.83–2.98	0.157
Physicians as source of information about diabetes	1.26	0.34	0.74–2.14	0.399
Employment status	0.95	0.32	0.49–1.83	0.876
Medication use	1.01	0.37	0.49–2.09	0.971

Model 2: risk perception for developing diabetes	Coeff.	SE	95% CI	*p* value

Perception of personal health status	−0.13	0.03	−0.19 to 0.08	<0.001
Who need additional information about diabetes	0.16	0.03	0.11–0.22	<0.001
Educational level				
College degree or higher	1^*∗*^			
No formal education/elementary/middle school	−0.17	0.04	−0.25 to 0.08	<0.001
High school	−0.1	0.04	−0.19 to 0.02	0.014
Who have a higher BMI	0.01	0.004	0.004–0.02	0.002
Female	0.09	0.03	0.03–0.14	0.003
Who have close relatives with diabetes	0.07	0.03	0.01–0.12	0.020
Age > 40	0.07	0.03	0.006–0.12	0.032
Who have at least a chronic disease	0.07	0.03	0.002–0.13	0.044
Who have more than one child	0.06	0.04	−0.02 to 0.13	0.134
Medication use	0.02	0.04	−0.05 to 0.09	0.587
Knowledge of the main risk and protective factors of diabetes	0.006	0.04	−0.07 to 0.09	0.875
Marital status	−0.001	0.04	−0.09 to 0.08	0.973

Model 3: worry of getting diabetes	Coeff.	SE	95% CI	*p* value

Educational level				
College degree or higher	1^*∗*^			
No formal education/elementary/middle school	0.36	0.1	0.16–0.56	0.001
High school	0.2	0.09	0.02–0.38	0.033
Who need additional information about diabetes	0.17	0.06	0.04–0.3	0.010
Who have a higher BMI	0.01	0.009	−0.002 to 0.03	0.089
Perception of personal health status	−0.11	0.07	−0.24 to 0.02	0.096
Age ≤ 40	−0.11	0.07	−0.26 to 0.03	0.133
Employment status	−0.11	0.08	−0.28 to 0.05	0.177
Female	0.09	0.08	−0.07 to 0.25	0.281
Who have more than one child	0.09	0.09	−0.09 to 0.27	0.315
Knowledge of the main risk and protective factors of diabetes	−0.07	0.09	−0.25 to 0.12	0.477

^*∗*^Reference category.

## Data Availability

The data used to support the findings of this study are available from the corresponding author upon request.
